# Feline Immunodeficiency Virus Evolutionarily Acquires Two Proteins, Vif and Protease, Capable of Antagonizing Feline APOBEC3

**DOI:** 10.1128/JVI.00250-17

**Published:** 2017-05-12

**Authors:** Rokusuke Yoshikawa, Junko S. Takeuchi, Eri Yamada, Yusuke Nakano, Naoko Misawa, Yuichi Kimura, Fengrong Ren, Takayuki Miyazawa, Yoshio Koyanagi, Kei Sato

**Affiliations:** a Laboratory of Viral Pathogenesis, Institute for Virus Research, Kyoto University, Kyoto, Japan; b Department of Bioinformatics, Medical Research Institute, Tokyo Medical and Dental University, Tokyo, Japan; c Laboratory of Signal Transduction, Institute for Virus Research, Kyoto University, Kyoto, Japan; d Laboratory of Virolution, Institute for Virus Research, Kyoto University, Kyoto, Japan; e CREST, Japan Science and Technology Agency, Saitama, Japan; University of Illinois at Chicago

**Keywords:** APOBEC3, FIV, Vif, evolutionary arms race, lentivirus, protease

## Abstract

The interplay between viral and host proteins has been well studied to elucidate virus-host interactions and their relevance to virulence. Mammalian genes encode apolipoprotein B mRNA-editing enzyme catalytic polypeptide-like 3 (APOBEC3) proteins, which act as intrinsic restriction factors against lentiviruses. To overcome APOBEC3-mediated antiviral actions, lentiviruses have evolutionarily acquired an accessory protein, viral infectivity factor (Vif), and Vif degrades host APOBEC3 proteins via a ubiquitin/proteasome-dependent pathway. Although the Vif-APOBEC3 interaction and its evolutionary significance, particularly those of primate lentiviruses (including HIV) and primates (including humans), have been well investigated, those of nonprimate lentiviruses and nonprimates are poorly understood. Moreover, the factors that determine lentiviral pathogenicity remain unclear. Here, we focus on feline immunodeficiency virus (FIV), a pathogenic lentivirus in domestic cats, and the interaction between FIV Vif and feline APOBEC3 in terms of viral virulence and evolution. We reveal the significantly reduced diversity of FIV subtype B compared to that of other subtypes, which may associate with the low pathogenicity of this subtype. We also demonstrate that FIV subtype B Vif is less active with regard to feline APOBEC3 degradation. More intriguingly, we further reveal that FIV protease cleaves feline APOBEC3 in released virions. Taken together, our findings provide evidence that a lentivirus encodes two types of anti-APOBEC3 factors, Vif and viral protease.

**IMPORTANCE** During the history of mammalian evolution, mammals coevolved with retroviruses, including lentiviruses. All pathogenic lentiviruses, excluding equine infectious anemia virus, have acquired the *vif* gene via evolution to combat APOBEC3 proteins, which are intrinsic restriction factors against exogenous lentiviruses. Here we demonstrate that FIV, a pathogenic lentivirus in domestic cats, antagonizes feline APOBEC3 proteins by both Vif and a viral protease. Furthermore, the Vif proteins of an FIV subtype (subtype B) have attenuated their anti-APOBEC3 activity through evolution. Our findings can be a clue to elucidate the complicated evolutionary processes by which lentiviruses adapt to mammals.

## INTRODUCTION

Viruses undergo drastic changes in virulence in response to alterations to their genetic information and cross-species transmission events, and elucidating the coevolutionary relationships between viruses and their hosts remains one of the most important undertakings in the fields of virology and evolutionary biology. For example, infection with a human lentivirus, human immunodeficiency virus type 1 (HIV-1), results in AIDS, while simian immunodeficiency viruses (SIVs), the evolutionary relatives of HIV-1, naturally infect more than 40 species of Old World monkeys (OWMs) in Africa but cause no known disorders (1–4). However, it has been challenging to address the virus-host coevolutionary history of SIVs because of the higher rates of evolution and frequent cross-species swapping of these viruses, and little is known about the factors determining and/or related to their pathogenicity.

To elucidate virus-host evolutionary relationships and their relevance to viral virulence, the interactions between viral and host proteins have been investigated. Certain cellular proteins have been identified to be barriers that impair cross-species infection and subsequent viral adaptation to hosts; these proteins are known as restriction or intrinsic immunity factors (5). Human apolipoprotein B mRNA-editing enzyme catalytic polypeptide-like 3 (APOBEC3) proteins (A3 proteins), particularly A3G, are cellular DNA cytosine deaminases and restriction factors that inhibit HIV-1 replication (6–8). Human A3 proteins are packaged into nascent HIV-1 particles and insert G-to-A hypermutations into newly synthesized viral cDNA, resulting in the abrogation of viral replication. Mammals have multiple *A3* genes in their genomes (9, 10), suggesting that A3-mediated restriction of lentiviral infection is universal across mammals. Mammalian *A3* genes are highly diverse and undergo positive selection (11, 12), allowing us to infer the evolution of mammalian *A3* genes to control lentiviral replication through an evolutionary arms race with lentiviruses.

To overcome human A3-mediated restriction, an HIV-1-encoded protein, viral infectivity factor (Vif), recruits the cellular ubiquitin E3 ligase complex and degrades A3 proteins via a ubiquitin/proteasome-dependent pathway, thereby impeding A3 packaging into nascent viral particles (6, 7). Although the *A3G* gene in OWMs is highly diversified, the Vif proteins encoded by SIVs have also evolved the ability to degrade the A3G proteins of their natural hosts (13–15), suggesting that Vif is required to exclude A3-mediated intrinsic host defenses against lentiviruses. Moreover, all lentiviruses, with the exception of equine infectious anemia virus, encode the *vif* gene in their genomes (16), and these lentiviral Vif proteins counteract A3-mediated antiviral actions in their hosts (17). Thus, A3 proteins, particularly those in primates, such as humans and OWMs, appear to facilitate robust activity against lentivirus infection, and the lentiviral Vif protein is crucial in allowing the virus to overcome A3-mediated restriction by the host.

Feline immunodeficiency virus (FIV), a feline lentivirus, was first discovered in 1987 in domestic cats (Felis catus) exhibiting AIDS-like symptoms (18). The genomes of domestic cats encode multiple *A3* genes, specifically, three *A3Z2* genes and a single *A3Z3* gene. Additionally, *A3Z2Z3*, an alternative splicing product of *A3Z2* and *A3Z3*, is endogenously expressed (19). The feline A3 proteins harboring the Z3 domain, A3Z3 and A3Z2Z3, potently abrogate infection caused by FIV strains in which *vif* is deleted (19–22). Similar to the interplay between primate A3 and lentiviral Vif, FIV Vif antagonizes the antiviral activity of feline A3 proteins by degrading these proteins in virus-producing cells (19–22). Moreover, FIV is classified into 4 subtypes, subtypes A to D, and viral pathogenicity differs among these viral subtypes; in particular, FIV subtype B is relatively less pathogenic than the other subtypes (23, 24). However, the viral factors determining FIV pathogenicity remain unknown.

In this study, we show that the genetic diversity of FIV subtype B is significantly lower than those of the other subtypes. Consistent with previous assumptions (23, 24), this finding implies a lower degree of pathogenicity of FIV subtype B. Additionally, the Vif proteins of FIV subtype B poorly antagonize feline A3 proteins. Phylogenetic and experimental approaches revealed that the Vif proteins of FIV subtype B had become attenuated in their ability to counteract feline A3 after the divergence from the other FIV subtypes. Furthermore, the FIV protease (PR) cleaves feline A3Z2Z3 in released virions. This is the first study to identify two types of anti-A3 factors, Vif and viral protease, encoded by a lentivirus genome.

## RESULTS

### FIV subtype B is less diversified than the other FIV subtypes.

**FIG 1:**
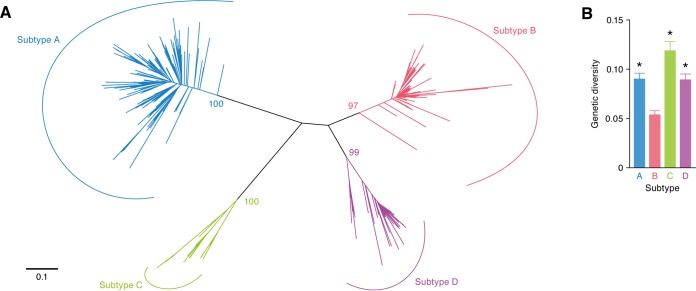
Less diversity of FIV subtype B. The sequences of the V3-V5 region of FIV *env* (subtype A, *n* = 153; subtype B, *n* = 100; subtype C, *n* = 24; subtype D, *n* = 49) were extracted from the GenBank/EMBL/DDBJ sequence database (the accession numbers used are available upon request). (A) Phylogenetic tree of FIV *env*. This phylogenetic tree was constructed using the ML method and displays an evolutionary relationship among the FIV sequences used in this study. Each line, called a “branch” of the tree, represents one FIV sequence. The longer branch lengths correlate with larger accumulations of mutations in the sequences. The scale bar indicates 0.1 nucleotide substitution per site. The bootstrap values are indicated on each node. (B) Viral genetic diversity. These values were calculated as described in Materials and Methods. Statistical analyses were performed using Bonferroni's multiple-comparison test. *, *P* < 0.01 versus subtype B.

Given the apparent inverse correlation between viral pathogenicity and viral diversity (25), we analyzed FIV diversity. We extracted 326 sequences of the V3-V5 region of FIV *env* from the GenBank/EMBL/DDBJ sequence database. In accordance with the findings of previous studies (26, 27), a phylogenetic tree classified FIV into 4 subtypes (Fig. 1A). Interestingly, genetic diversity analyses revealed that the *env* gene of FIV subtype B was less diverse than the *env* genes of the other subtypes (Fig. 1B).

### The anti-feline A3 activity of FIV subtype B Vif is attenuated.

**FIG 2:**
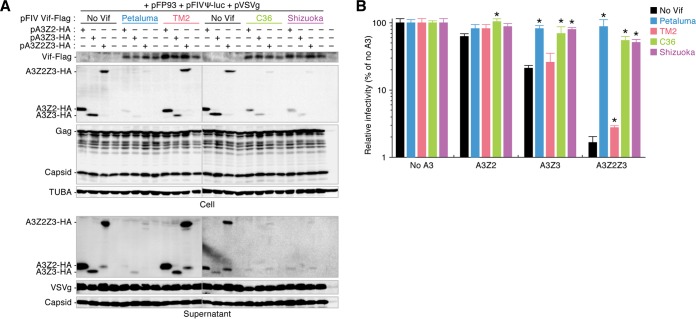
Attenuated degradation activity of FIV subtype B Vif against feline A3. The expression plasmids for Flag-tagged FIV Vif were cotransfected with or without the expression plasmids for feline A3Z2, A3Z3, and A3Z2Z3 tagged with HA. Representative results of Western blotting assays (A) and FIV reporter assays (B) are shown. * in panel B, *P* < 0.05 versus no Vif. The assays were independently performed in triplicate. The data represent averages with SDs.

As viral pathogenicity and diversity are closely associated with the interactions between viral proteins and host restriction factors (28–30), we hypothesized that the relatively low level of diversity of FIV subtype B (Fig. 1) was attributable to the inability of viral proteins to antagonize feline restriction factors. According to previous reports (19–22), feline A3 proteins, particularly A3Z3 and A3Z2Z3, strongly impair FIV infection, whereas FIV Vif counteracts the feline A3-mediated antiviral actions. Moreover, a previous report has implied that the anti-feline A3 actions of FIV Vif were associated with viral replication capacity and pathogenicity (31). Therefore, we investigated the ability of the Vif protein of each FIV subtype to degrade feline A3 proteins in cell-based experiments. Feline A3 expression plasmids and pFP93 (a packaging plasmid in which FIV *vif* is deleted) (32) were cotransfected with or without a plasmid expressing the Vif of each FIV subtype (strain Petaluma for subtype A, strain TM2 for subtype B, strain C36 for subtype C, and strain Shizuoka for subtype D). In the absence of FIV Vif, all feline A3 proteins, specifically A3Z2, A3Z3, and A3Z2Z3, were efficiently incorporated into the released viral particles (Fig. 2A). Consistent with the findings of previous studies (19–22), feline A3Z3 and A3Z2Z3, but not A3Z2, significantly suppressed the infectivity of FIV from which *vif* was deleted (Fig. 2B). In the presence of the Vif proteins of FIV subtypes A (strain Petaluma), C (strain C36), and D (strain Shizuoka), feline A3 proteins were degraded and packaging into virions was impaired (Fig. 2B). In contrast, none of the feline A3 proteins were completely degraded by the Vif of FIV subtype B (strain TM2), and the A3 proteins were incorporated into released viral particles at levels comparable to those achieved in the absence of Vif (Fig. 2A). Moreover, the Vif of FIV subtype B (strain TM2) was incapable of neutralizing the suppression of FIV infectivity by feline A3Z3 and A3Z2Z3 (Fig. 2B).

**FIG 3:**
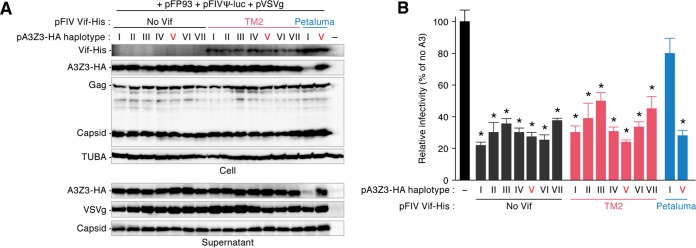
Effect of FIV Vif against feline A3Z3 haplotypes. The expression plasmids for His-tagged Vif of FIV TM2 or FIV Petaluma were cotransfected with or without the expression plasmids for a series of feline A3Z3 haplotypes tagged with HA. Representative results of Western blotting assays (A) and FIV reporter assays (B) are shown. * in panel B, *P* < 0.05 versus no A3Z3. The assays were independently performed in triplicate. The data represent averages with SDs. Note that the feline A3Z3 used in the other experiments is haplotype I.

Given the unique phenotype of the FIV subtype B (strain TM2) Vif (Fig. 2), it is possible that this protein has acquired other abilities. In this regard, a previous report described the polymorphism of the *A3Z3* gene in domestic cats (33), and importantly, we have recently demonstrated a haplotype of feline A3Z3, haplotype V, that is resistant to degradation mediated by the Vif proteins of FIV subtypes A (strain Petaluma), C (strain C36), and D (strain Shizuoka) (22). Based on these observations, we hypothesized that the FIV subtype B (strain TM2) Vif uniquely acquired the ability to degrade feline A3Z3 haplotype V. However, the TM2 (subtype B) Vif was unable to degrade A3Z3 haplotype V (Fig. 3A), similar to the findings for Petaluma (subtype A) Vif. Additionally, all A3Z3 haplotypes were incorporated into the released virions (Fig. 3A) and suppressed FIV infectivity even in the presence of the TM2 (subtype B) Vif (Fig. 3B). Therefore, these results argue against the possibility that the FIV subtype B (strain TM2) Vif lost its ability to degrade a conventional feline A3Z3 (haplotype I) in exchange for an antagonizing ability against other A3Z3 haplotypes, including haplotype V.

**FIG 4:**
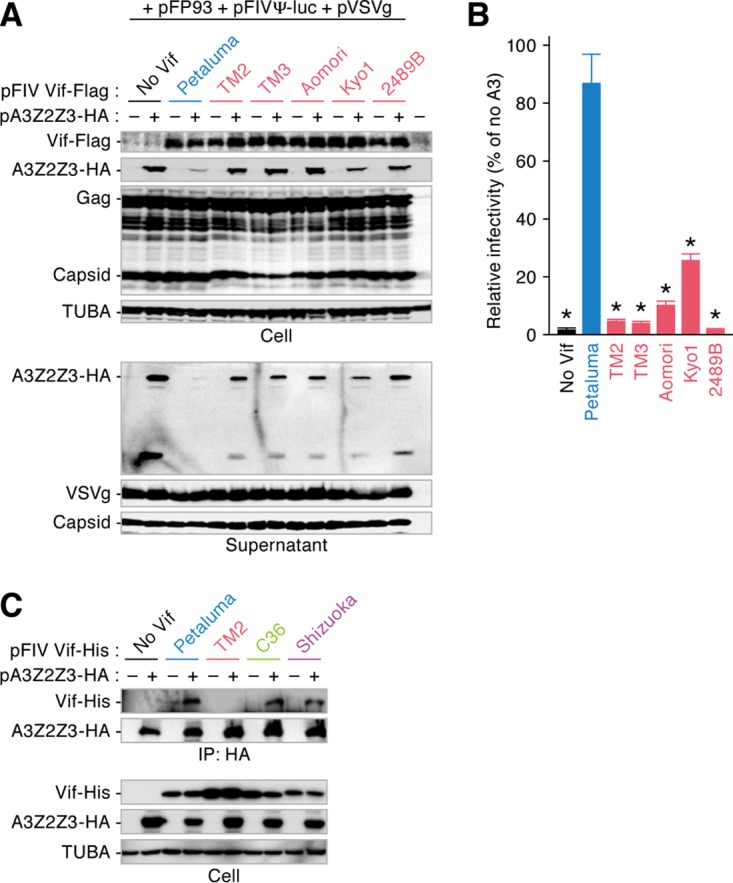
Conservation of reduced degradation activity of FIV subtype B Vif against feline A3. The expression plasmids for Flag-tagged FIV Vif were cotransfected with or without the expression plasmids for feline A3Z2Z3 tagged with HA. Representative results of Western blotting assays (A) and FIV reporter assays (B) are shown. FIV infectivity is shown as the percentage of the value for no A3Z2Z3. * in panel B, *P* < 0.05 versus Petaluma. The assays were independently performed in triplicate. Data represent averages with SDs. (C) Co-IP assay. The expression plasmids for His-tagged FIV Vif were cotransfected with or without the expression plasmids for feline A3Z2Z3 tagged with HA, and the co-IP assay was performed as described in Materials and Methods. Representative results from co-IP assays using an anti-HA antibody (top) and cell lysate (bottom) are shown.

To address whether the attenuated ability of FIV Vif to degrade feline A3 is conserved in subtype B, we prepared Vif expression plasmids for 4 additional strains of FIV subtype B: strains TM3, Aomori, Kyo1, and 2489B. In particular, we determined the *vif* sequences of FIV strains Aomori and Kyo1, which are classified into subtype B (26). As shown in Fig. 4A, feline A3Z2Z3 was not degraded by any of the tested FIV subtype B Vif proteins, whereas it was degraded by the FIV subtype A Vif protein (strain Petaluma), and feline A3Z2Z3 proteins were incorporated into the released virions. Although the Vif of FIV subtype A (strain Petaluma) significantly counteracted the antiviral activity of feline A3Z2Z3 by inhibiting packaging, the infectivity of FIV released from the cells expressing FIV subtype B Vif was significantly lower than that of FIV released from the cells expressing FIV subtype A (strain Petaluma) (Fig. 4A and B). Thus, the inability of FIV Vif to degrade the feline A3 protein is a unique and maintained feature of subtype B.

Next, to assess the mechanism by which subtype B Vif lost the ability to degrade feline A3 proteins, we performed a coimmunoprecipitation (co-IP) assay with FIV Vif and feline A3. As shown in Fig. 4C, the feline A3Z2Z3 protein precipitated with the Vif proteins of subtypes A (strain Petaluma), C (strain C36), and D (strain Shizuoka). In contrast, the Vif of subtype B (strain TM2) faintly bound to feline A3Z2Z3 (Fig. 4C). Therefore, these results suggest that the reduced ability of the subtype B Vif to bind to feline A3 results in an inability to degrade feline A3 proteins.

### Residue 167 determines the attenuated ability of FIV subtype B Vif to degrade feline A3 protein.

**FIG 5:**
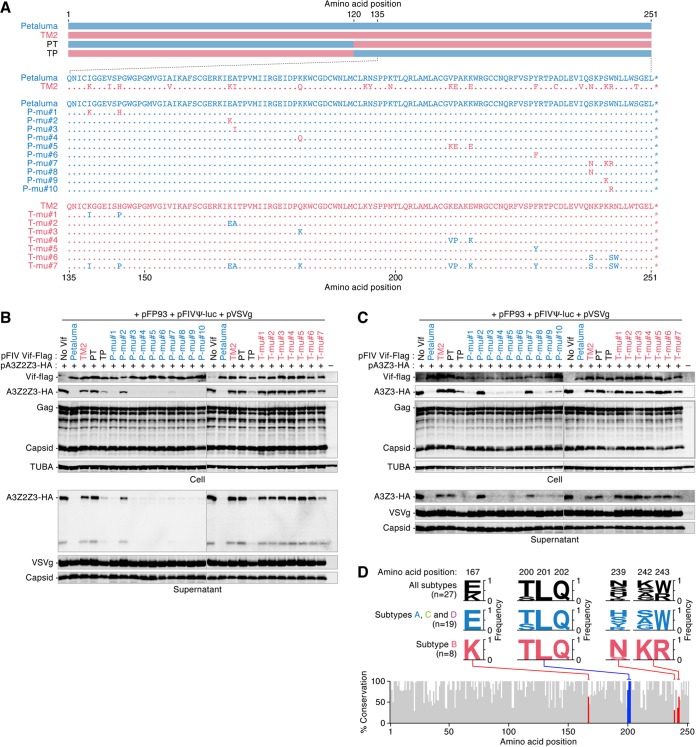
The responsive residue determines the ability of FIV Vif to degrade feline A3. (A) Schematic depicting the FIV Vif derivatives used. (B and C) Expression plasmids for a series of FIV Vif derivatives tagged with Flag were cotransfected with or without the expression plasmids for feline A3Z2Z3 (B) or A3Z3 (C) tagged with HA. Representative results of Western blotting assays are shown. (D) VESPA analysis. The percent amino acid sequence conservation was analyzed using VESPA (http://www.hiv.lanl.gov/content/sequence/VESPA/vespa.html) (34). The accession numbers of the FIV Vif sequences (subtype A, *n* = 5; subtype B, *n* = 8; subtype C, *n* = 10; subtype D, *n* = 4) are listed in Table 1. In the bottom panel, residues 167 and 242 to 243 are indicated in red, while the (T/S)LQ motif (residues 200 to 202) is indicated in blue. In the top panel, the amino acids in these positions are indicated as logo plots. *n*, the number of sequences used.

To identify the region of FIV Vif that determines the ability to degrade the feline A3 protein, we constructed two types of Vif proteins, swapping derivatives based on FIV Petaluma (subtype A) and TM2 (subtype B), which were designated the PT and TP derivatives, respectively (Fig. 5A, top). As shown in Fig. 5B and C, the TP derivative degraded feline A3Z2Z3 and A3Z3, while the PT derivative did not. Additionally, the TP derivative impaired the incorporation of A3Z2Z3 (Fig. 5B) and A3Z3 (Fig. 5C) into released viral particles, but the PT derivative did not. These results suggest that the C-terminal region of FIV Vif determines the ability to degrade feline A3 proteins.

To further identify the responsible amino acid(s) at the single residue level, a series of Vif mutants was constructed (Fig. 5A, bottom). As shown in Fig. 5B, 9 out of the 10 derivatives of FIV Petaluma (subtype A) Vif degraded feline A3Z2Z3, while the P-mu#2 derivative, which contains a substitution of glutamic acid to lysine at position 167 (E167K), lost the ability to degrade feline A3Z2Z3. These results suggest that the residue at position 167 in FIV Vif determines the ability of this protein to degrade feline A3Z2Z3. We next assessed the ability of these Petaluma Vif derivatives to degrade feline A3Z3. Interestingly, P-mu#2 and another derivative, P-mu#7 (S239N/S242K/W243R), were unable to degrade feline A3Z3 (Fig. 5C). Because the three derivatives P-mu#8 (S239N), P-mu#9 (S242K), and P-mu#10 (W243R) degraded feline A3Z3 (Fig. 5C), these three residues at positions 239, 242, and 243 were crucial for the degradation of A3Z3. Conversely, we further prepared seven Vif derivatives based on the FIV TM2 (subtype B) Vif. In contrast to the results obtained with the Petaluma derivatives, none of the TM2 derivatives was able to degrade feline A3Z2Z3 (Fig. 5B) and A3Z3 (Fig. 5C). These results suggest a high degree of constraint on the attenuated activity of subtype B Vif.

To further assess differences in the Vif sequence between subtype B and other subtypes, we analyzed the identity of the FIV Vif from each subtype (the accession numbers of the *vif* sequences are listed in Table 1). The identities of the amino acid sequences of FIV Vif among the subtypes ranged from 80.1 to 87.3%, suggesting that the sequence of the subtype B Vif does not significantly differ from the Vif sequences of the other subtypes. Next, we aligned the Vif sequences of 27 FIV strains and analyzed the conservation of each residue using an application called VESPA (http://www.hiv.lanl.gov/content/sequence/VESPA/vespa.html) (34). As shown in Fig. 5D, the (S/T)LQ motif, which is crucial for binding to the cellular E3 ligase complex (21), was completely conserved in all tested FIV Vif sequences. Intriguingly, the Vif proteins of subtypes A, C, and D encoded glutamic acid at position 167 (*n* = 19 in total), while the Vif proteins of subtype B (*n* = 8) encoded lysine at this position (Fig. 5D). Therefore, it is plausible to assume that the loss of function of FIV subtype B Vif to degrade feline A3Z3 (Fig. 5C) and A3Z2Z3 (Fig. 5B) is determined by the lysine at position 167, and this phenotype is evolutionarily conserved within this subtype. Moreover, the residues at positions 239, 242, and 243, which are closely associated with the ability to degrade feline A3Z3 (Fig. 5C), were highly conserved in FIV subtype B, while these residues were relatively divergent in subtypes A, C, and D (Fig. 5D). Therefore, these three residues may contribute to the evolutionary constraint on the attenuated activity of subtype B Vif for the degradation of feline A3Z3.

**TABLE 1:** Accession numbers for *vif* sequences

Subtype	Accession no.	Strain	No. of clones
A	M25381	Petaluma	
A	LC177510^*a*^	N91	1
A	LC177511^*a*^	N91	5
A	M36968	PPR	
A	X57002	SwissZ1	
B	LC177505^*a*^	Aomori	1
B	U11820	2489B	
B	LC177506^*a*^	Kyo1	1
B	LC179607^*a*^	TM1	
B	M59418	TM219	
B	LC177507^*a*^	TM3	1
B	LC177508^*a*^	TM3	2
B	LC177509^*a*^	TM3	4
C	AY600517	C36	
C	LC177497^*a*^	pcGammer	1
C	LC179608^*a*^	pcGammer	2
C	LC177498^*a*^	pcGammer	5
C	LC177499^*a*^	TI2	1
C	LC177500^*a*^	TI2	2
C	LC177501^*a*^	TI2	6
C	LC177502^*a*^	TI4	1
C	LC177503^*a*^	TI4	2
C	LC177504^*a*^	TI4	6
D	LC179609^*a*^	Shizuoka	1
D	LC179610^*a*^	Shizuoka	2
D	LC179611^*a*^	Shizuoka	3
D	LC179612^*a*^	Shizuoka	4

a The sequences newly identified in this study.

### The attenuated anti-feline A3 activity of FIV subtype B Vif is acquired and thus far maintained during evolution.

**FIG 6:**
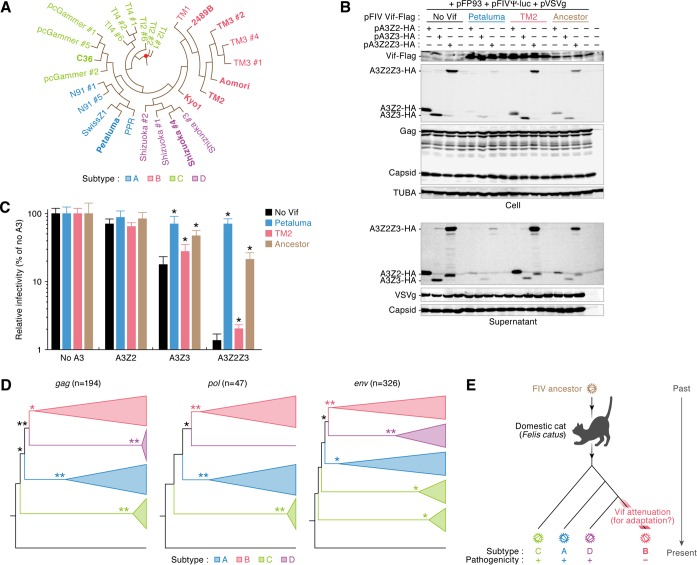
Evolution of FIV Vif. (A) Reconstructed phylogenetic tree of FIV *vif*. A red dot indicates the ancestral node of FIV *vif*. (B and C) Expression plasmids for Flag-tagged FIV Vif were cotransfected with or without the expression plasmids for feline A3Z2, A3Z3, and A3Z2Z3 tagged with HA. Representative results of Western blotting assays (B) and FIV reporter assays (C) are shown. * in panel C, *P* < 0.05 versus no A3. The assays were independently performed in triplicate. Data represent averages with SDs. (D) Phylogenetic trees of FIV genes. The phylogenetic trees of *gag*, *pol*, and *env* are shown. *n*, the number of sequences used. The bootstrap values are as follows: >50% (*) and >80% (**). An FIVpco sequence (GenBank accession no. KF906157) was specified as the root of these trees. (E) Schematic of putative scenario for FIV Vif evolution.

To investigate when and how FIV subtype B attenuated the function to degrade feline A3 proteins, we constructed a phylogenetic tree for FIV *vif*. On the basis of this phylogenetic tree (Fig. 6A), the ancestral sequence of FIV *vif* was estimated. We then prepared an expression plasmid for the estimated ancestor of FIV Vif and performed cell-based cotransfection experiments. As shown in Fig. 6B, the ancestral Vif degraded all feline A3 proteins and inhibited their incorporation into nascent virions. Additionally, the suppression of FIV infectivity by feline A3Z3 and A3Z2Z3 was neutralized by ancestral Vif (Fig. 6C). These results suggest that the ancestor of FIV Vif had the ability to antagonize feline A3-mediated antiviral action. Moreover, the phylogenetic trees constructed for three universal retroviral genes (*gag*, *pol*, and *env*) of FIV demonstrated similar topologies, and FIV subtype B was most recently separated from subtype D (Fig. 6D). Taken together, these findings suggest that the anti-feline A3 activity of FIV Vif subtype B was attenuated after divergence from subtype D and is thus far maintained (Fig. 6E). The lower genetic diversity of FIV subtype B (Fig. 1) may be a reflection of its younger age and more recent origins.

### FIV protease cleaves feline A3Z2Z3 in nascent viral particles.

**FIG 7:**
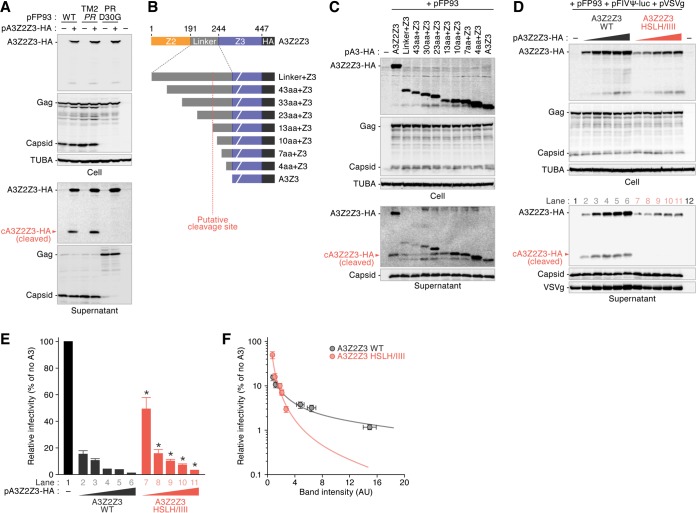
Cleavage of feline A3Z2Z3 by FIV PR. (A) Expression plasmids for feline A3Z2Z3 tagged with HA were cotransfected with pFP93 derivatives. Representative results of Western blotting assays are shown. Cleaved feline A3Z2Z3 (cA3Z2Z3) in the released viral particle is indicated with red arrowheads. (B) Schematic of the feline A3Z2Z3 derivatives used in the assay whose results are presented in panel C. The putative cleavage site is indicated with a vertical broken red line. (C) Expression plasmids for feline A3Z2Z3 derivatives tagged with HA were cotransfected with pFP93. Representative results of Western blotting assays are shown. (D to F) Expression plasmids for feline A3Z2Z3 WT or HSLH/IIII mutant derivatives (0, 200, 400, 800, 1,200, and 1,800 ng) were cotransfected with pFP93 and FIV-based virus plasmids. Representative results of Western blotting assays (D) and FIV reporter assays (E) are shown. * in panel E, *P* < 0.05 versus A3Z2Z3 WT. The assays were independently performed in triplicate. The data represent averages with SDs. The lane numbers in panel D correlate with those in panel E. (F) A correlation between the amount of A3Z2Z3 and cleaved A3Z3 (cA3Z3) in the released viral particle (*x* axis) and the viral infectivity (*y* axis) is shown. The circles with error bars represent averages with SDs (*n* = 3), and the lines represent exponential approximation. AU, arbitrary units.

Here we demonstrated that the Vif proteins of FIV subtype B commonly lost the ability to antagonize feline A3 proteins (Fig. 4). However, FIV subtype B circulates in the wild (26, 35–37), and strains of this subtype, including strain TM2, infect and replicate in certain feline cell lines (e.g., MYA-1 cells) expressing endogenous *A3* genes (38). Our findings, together with previous observations, suggest the ability of FIV to circumvent feline A3-mediated antiviral effects independently of Vif. In this regard, Abudu et al. reported that the murine A3 protein is cleaved in the virion by the viral protease (PR) of murine leukemia virus (MLV) (39). Although the molecular mass of hemagglutinin (HA)-tagged feline A3Z2Z3 is approximately 50 kDa, a protein approximately 25 kDa in size was also detected in the supernatant blots (Fig. 2A, 4A, 5B, and 6B). Based on these findings, we assumed that FIV PR possesses the ability to cleave feline A3Z2Z3. To address this issue, we constructed a plasmid carrying a defective FIV PR based on pFP93 and designated this plasmid PR D30G (Fig. 7A). Consistent with a previous report (40), the PR D30G derivative abolished the cleavage of the Gag precursor (Fig. 7A). In addition, the 25-kDa protein derived from feline A3Z2Z3 was not detected in the culture supernatant (Fig. 7A, bottom), suggesting that FIV PR cleaves feline A3Z2Z3 in released virions in an enzymatic activity-dependent manner.

The pFP93 plasmid is based on FIV subtype A (strain Petaluma), and the amino acid sequence identity between the PRs of Petaluma and TM2 (subtype B) is 90.0%. To investigate whether the PR of subtype B FIV cleaves feline A3Z2Z3, we next constructed a plasmid, pFP93prTM2 (indicated TM2 *PR* in Fig. 7A), which is a pFP93 derivative containing the PR of TM2 (subtype B). As shown in Fig. 7A, the levels of cleaved A3Z2Z3 (cA3Z2Z3) in the viral particles of TM2 *PR* were comparable to those in the viral particles of parental pFP93. Thus, FIV PR cleaves feline A3Z2Z3 in nascent viral particles regardless of subtype.

We then addressed the cleavage site of feline A3Z2Z3 by FIV PR. Feline A3Z2Z3 consists of the Z2, linker, and Z3 regions (Fig. 7B, top). Because the molecular mass of cA3Z2Z3 is approximately 25 kDa, the FIV PR cleavage site of feline A3Z2Z3 is predicted to be located in the linker region. To determine the cleavage site of feline A3Z2Z3, we prepared a set of feline A3Z2Z3 truncated mutants (Fig. 7B, bottom). As shown in Fig. 7C, the molecular mass of cA3Z2Z3 was similar to the molecular masses of the 13aa+A3, 10aa+A3, and 7aa+A3 mutants, implying a cleavage site location between positions 222 and 232 in feline A3Z2Z3.

As a previous study suggested the importance of leucine for cleavage site recognition by retroviral PR (41), we assumed that the leucine residue at position 232 of feline A3Z2Z3 was related to cleavage by FIV PR. To investigate the effect of FIV PR-mediated A3Z2Z3 cleavage on viral infectivity, we constructed a plasmid expressing a feline A3Z2Z3 derivative in which the histidine, serine, leucine, and histidine residues at positions 230 to 233 were replaced by isoleucines (A3Z2Z3 HSLH/IIII). Although the expression level of the HSLH/IIII mutant was lower than that of the wild-type (WT) A3Z2Z3 protein, no cA3Z2Z3 was detected in the released viral particles (Fig. 7D). These findings suggest that FIV PR cleaves feline A3Z2Z3 by recognizing the amino acids at positions 230 to 233. We then evaluated the infectivity of the released virions and found that the HSLH/IIII mutant suppresses viral infectivity significantly less than the A3Z2Z3 WT (Fig. 7E). However, the amount of HSLH/IIII protein encapsidated in the viral particles was clearly less than that of the A3Z2Z3 WT protein (Fig. 7D, bottom). For instance, although the infectivity of lane 11 (HSLH/IIII, 1,800 ng) was comparable to those of lanes 4 and 5 (WT, 800 and 1,200 ng, respectively) (Fig. 7E), the band intensity of A3Z2Z3 in the supernatant of lane 11 was clearly lower than those in the supernatant of lanes 4 and 5 (Fig. 7F). Therefore, to quantitatively evaluate these results, we normalized viral infectivity (Fig. 7E) to the amount of feline A3 proteins detected in the supernatant blot (Fig. 7D, bottom). As shown in Fig. 7F, we revealed that the antiviral activity of the HSLH/IIII mutant was more robust than that of the A3Z2Z3 WT. Altogether, these findings suggest that FIV PR-mediated cleavage of feline A3Z2Z3 potently contributes to counteracting the antiviral effect mediated by feline A3Z2Z3.

## DISCUSSION

In this study, we demonstrated the uniquely attenuated ability of FIV subtype B to degrade feline A3 proteins (Fig. 2 to 4). As our findings assume that ancestral FIV Vif possesses anti-feline A3 abilities (Fig. 6), subtype B Vif appears to have obtained attenuated anti-feline A3 activity during evolution after divergence from other subtypes. Moreover, FIV PR cleaves feline A3Z2Z3 in released virions, and this PR activity is conserved in all FIV subtypes tested (Fig. 7). In summary, this is the first study to describe a lentivirus whose genome encodes two types of anti-A3 factors, Vif and PR.

Viral diversity is closely associated with viral pathogenicity. For example, the genetic diversity of SIVs that are apathogenic in OWMs, the natural hosts of SIVs in the wild, is relatively low because these viruses do not undergo immune pressures (25). In this study, molecular phylogenetic analyses revealed that the genetic diversity of the *env* gene of FIV subtype B was significantly lower than that of the *env* genes of the other subtypes (Fig. 1). This implies that the pathogenicity of FIV subtype B is relatively lower than that of the other subtypes. Our findings are related to the observations made in previous epidemiological studies: all FIVs detected in Brazil are subtype B (23, 42, 43), and the prevalence of FIV in domestic cats in that country is relatively high (approximately 27%). Nevertheless, the cats infected with FIV in Brazil do not exhibit any remarkable disorders (44, 45), and previous studies have suggested that FIV subtype B is less virulent than the other subtypes (23, 24). Indeed, domestic cats experimentally infected with FIV strains TM1 and TM2 (subtype B) were asymptomatic for over 8 years, while infection with FIV strain Petaluma (subtype A) resulted in severe AIDS-like disorders (46). Therefore, FIV subtype B appears to be relatively less pathogenic. Moreover, cell-based experiments have revealed that the level of growth of FIV strain TM2 in a feline CD4^+^ T-cell line expressing endogenous *A3* genes is significantly lower than that of strain Petaluma (19, 38), and this difference in viral growth is determined by specific viral genes, such as *gag*, *pol*, and *vif* (38). The relatively lower replication capacity of FIV strain TM2 in cells expressing A3 is likely attributable to the attenuation of Vif. In this study, the anti-feline A3 activities of the Vif proteins of all 5 subtype B strains tested were significantly lower than the activity of the Vif protein of subtype A (strain Petaluma) (Fig. 4), and the ancestral FIV Vif antagonized feline A3 (Fig. 6B and C). Taken together, these findings highlight a unique and conserved phenotype of FIV subtype B Vif, which is characterized by an attenuated ability to degrade feline A3. Furthermore, our results suggest that this phenotype of FIV subtype B Vif was acquired during the evolution of subtype B after it diverged from other FIV subtypes (Fig. 6E). In contrast to FIV subtype B Vif, the Vif proteins of apathogenic SIVs maintain active antagonism of A3 in their respective hosts (13, 14). Therefore, FIV subtype B might be less pathogenic through molecular mechanisms different from those in SIVs.

Why has FIV subtype B attenuated its ability to counteract feline A3? If an anti-feline A3 ability is indispensable for FIV infection, subtype B FIV should have reacquired this ability. For example, during natural SIV infections in OWMs, SIV Vif possesses the ability to degrade host A3G proteins (13, 14). There are three possible explanations for this phenomenon: first, feline A3 may not be required to overcome efficient FIV replication. However, previous reports argue against this possibility because FIV from which *vif* was deleted is unable to replicate in both *in vitro* cell culture (47) and *in vivo* (48), suggesting the strong antiviral effects of feline endogenous A3 proteins. Second, the attenuation of anti-feline A3 activity may result in the failure of FIV subtype B to achieve optimal adaptation in domestic cats, and this subtype is decreasing within the cat population. This assumption is reminiscent of the insights into HIV-1; HIV-1 group M (major or main) is a global pandemic strain, while other HIV-1 groups are endemic in Africa. Thus, unsuccessful adaptation of these viruses to the human population has been postulated (49, 50). However, FIV subtype B has been detected in Japan (26, 35, 36), North America (51, 52), South America (23, 42, 43, 53), and Europe (37, 54, 55), suggesting the successful worldwide spread of FIV subtype B. Therefore, it is not plausible that subtype B is decreasing. Third, FIV subtype B Vif may purposefully maintain its attenuated ability. The attenuation of the viral pathogenicity of FIV subtype B is reminiscent of that of myxoma virus in European rabbits (Oryctolagus cuniculus) in England and Australia (56, 57). Because the lethality of myxoma virus infection in European rabbits was greater than 99%, this virus was imported into Australia to exterminate wild rabbits in the 1950s (58). However, this virus lost its pathogenicity within a few years (58). Phenomena in which less-virulent viruses are more advantageous than highly virulent viruses have been explained theoretically (56, 59). Furthermore, the attenuation of myxoma virus in Australia is attributable to the acquisition of a mutation in a viral gene, M156R (29). The M156R protein of the parental myxoma virus acts as an antagonist against the rabbit protein kinase R, which potently inhibits myxoma virus replication, while the mutated M156R protein of the attenuated virus does not (29). Thus, myxoma virus lost the ability to antagonize the host protein kinase R, attenuating its virulence and allowing it to adapt to the host. In this study, FIV subtype B lost the ability to degrade feline A3. Similar to the case of myxoma virus infection in rabbits, the loss of anti-feline A3 activity appears to be a strategy of FIV subtype B to attenuate its pathogenicity, leading to adaptation to its natural host. Supporting this concept, our mutagenesis experiments did not identify any gain-of-function derivatives of subtype B Vif to antagonize feline A3. Alternatively, a substitution mutant of subtype A Vif, the E167K (P-mu#2) mutant, was detected as a loss-of-function derivative to degrade feline A3Z2Z3 and A3Z3 (Fig. 5B), while another derivative, the S239N/S242K/W243R (P-mu#7) mutant, lost the ability to degrade feline A3Z3 (Fig. 5C). Furthermore, the residues at positions 167, 239, 242, and 243, which determine the degradation ability of feline A3, were highly conserved in subtype B Vif (Fig. 5D). Taking these findings together, the attenuation of the anti-feline A3 function of Vif appears to be an evolutionary strategy permitting FIV subtype B to adapt to naturally infected domestic cats, and this unique characteristic has been conserved during the evolution of FIV subtype B.

In addition to the unique characteristics of FIV subtype B Vif, FIV PR cleaves the feline A3Z2Z3 protein into viral particles (Fig. 7). This A3 cleavage is reminiscent of observations made with MLV, the genome of which does not encode *vif*; MLV PR excludes murine A3 in virions (39). Similarly, in our study, FIV PR cleaved virion-incorporated feline A3Z2Z3 in an enzymatic activity-dependent manner (Fig. 7A). Moreover, the PR of TM2 (subtype B) also cleaved feline A3Z2Z3 (Fig. 7A). These findings are correlated with observations made in a previous report in which FIV strain TM2 replicates in feline cell lines expressing endogenous *A3* genes, and feline A3 is poorly antagonized by Vif (38). The genomes of all lentiviruses, excluding equine infectious anemia virus, encode *vif*, and these lentiviruses are believed to have been evolutionarily endowed with *vif* to antagonize antiviral host A3 proteins, as lentiviral Vifs are the only factors that combat host A3 (17, 19, 20, 22, 60, 61). To the best of our knowledge, this report is the first to describe a lentivirus whose genome encodes at least two types of anti-A3 proteins, PR and Vif.

In summary, we identified two anti-A3 proteins, Vif and PR, encoded by the FIV genome. We also observed the attenuated ability of FIV subtype B Vif to antagonize feline A3-mediated antiviral action, and this attenuation was highly conserved in subtype B. Because the genetic diversity of FIV subtype B is significantly lower than that of the other subtypes, the attenuation of the anti-feline A3 activity of subtype B Vif appears to be a unique adaptation strategy employed by FIV to naturally infect domestic cats in the absence of virulence. This is the first study to propose the potential of lentiviruses to control their pathogenicity by attenuating the ability of Vif to antagonize the actions of host antiviral A3 proteins. Our findings shed light on the complicated evolutionary processes experienced by lentiviruses to permit adaptation to mammals.

## MATERIALS AND METHODS

### *vif* sequencing analyses for FIV isolates.

RNA was isolated from viral preparations of FIV strains N91, TI2, TI4, TM2, TM3, Aomori, and Kyo1 using a QIAamp viral RNA minikit (Qiagen). Reverse transcription (RT) was performed using SuperScript III reverse transcriptase (Thermo Fisher Scientific), and RT-PCR was performed using PrimeSTAR GXL DNA polymerase (TaKaRa) and the following primers: FIV vif Fwd (CAG TRT TAT TAA AGG ATG AAG AGA RGG G) and FIV vif Rev (CTA AAK GGG TTT AYT CCT GGR TTA ART GG). The resulting products were purified by gel extraction and then cloned using a Zero Blunt TOPO cloning kit (Thermo Fisher Scientific). Nucleotide sequences were determined by a DNA sequencing service (Fasmac), and the data were analyzed using Sequencher (v 5.1) software (Gene Codes Corporation). All FIV *vif* sequences were submitted to GenBank/EMBL/DDBJ.

### Phylogenetic and viral diversity analyses.

The sequences of the V3-V5 region of FIV *env* (*n* = 326) were obtained from the GenBank/EMBL/DDBJ sequence database and were aligned using the MAFFT program (62). The best-fitting substitution model was determined using the FindModel tool (63), and the Akaike information criterion selected general time-reversible (GTR) model with gamma distribution (G; GTR+G) as the best fit. Then, to identify the subtypes of 326 FIV *env* sequences, a phylogenetic tree was constructed using both the maximum likelihood (ML) method with the PhyML program (64) (GTR+G) and the neighbor-joining method with the MEGA6 program (65) (Tamura-Nei model+G). The two methods yielded tress with nearly identical topologies, and the ML tree is presented in Fig. 1A. The overall mean distance (genetic diversity in Fig. 1B) of the *env* V3-V5 sequences was calculated for each subtype with MEGA6 software employing the Tamura-Nei model, and the differences between subtypes were compared for statistical significance. To construct the phylogenetic trees shown in Fig. 6D, we retrieved FIV *gag* (*n* = 194), *pol* (*n* = 47), and *env* (*n* = 326) gene sequences from the GenBank/EMBL/DDBJ sequence database, and the accession numbers are available upon request. These genes were individually aligned with an FIV sequence derived from a puma (FIVpco; GenBank accession no. KF906157) using the MAFFT program (62). The *env* gene alignment had many gaps; therefore, the program Gblocks (66) was applied to further refine the *env* alignment. The ML method with PhyML (GTR+G) was employed to construct the trees for these FIV genes with 100 bootstrap replicates. The FIVpco sequence was specified as the root of each tree.

### Reconstruction of the ancestral sequence of FIV *vif*.

To infer the ancestral sequence of FIV *vif*, we constructed a phylogenetic tree of 27 FIV *vif* sequences using the ML method with PhyML and performed 100 bootstrap replicates. The accession numbers are listed in Table 1. The GTR+G substitution model was selected for tree construction by the use of the FindModel tool (63). Using this tree (Fig. 6A), the codeml program in the PAML package (67) was employed to infer the ancestral sequence. The root was assumed to be the midpoint of the tree.

### Plasmid construction.

HA-tagged feline A3 expression plasmids (based on pcDNA3.1) were kindly provided by Carsten Münk or prepared in our previous studies (22, 60, 68). To construct the expression plasmids for a series of feline A3 derivatives, the following primers were used: Linker+Z3 Fwd (AAA AAA GCT TGC CAC CAT GAG TCC CGG CCA ACA AAG AAA AAG), 43aa+Z3 Fwd (AAA AAA AGC TTG CCA CCA TGC CCT TCC CTC CCC GCC CAG G), 33aa+Z3 Fwd (AAA AAA AGC TTG CCA CCA TGG ACC CAA GGA GTT GGG TTC AG), 23aa+Z3 Fwd (AAA AAA AGC TTG CCA CCA TGG AGC CTG GGA TAA ACA CCA G), 13aa+Z3 Fwd (AAA AAA AGC TTG CCA CCA TGC TGC ACC TTT TGG TTT CCT TC), 10aa+Z3 Fwd (AAA AAA AGC TTG CCA CCA TGT TGG TTT CCT TCC TCT TGC C), 7aa+Z3 Fwd (AAA AAA AGC TTG CCA CCA TGT TCC TCT TGC CCA GAC CCA C), 4aa+Z3 Fwd (AAA AAA AGC TTG CCA CCA TGC CCA GAC CCA CAA TGA ATC C), Z2Z3 HSLH IIII Fwd (GAG CCT GGG ATA AAC ACC AGA AGA ATC ATT ATC ATC CTT TTG GTT TCC TTC C), Z2Z3 HSLH IIII Rev (GGA AGG AAA CCA AAA GGA TGA TAA TGA TTC TTC TGG TGT TTA TCC CAG GCT C), and BGH Rev (TAG AAG GCA CAG TCG AGG). Codon-optimized open reading frames of the *vif* genes of FIV strains Petaluma, TM2, C36, Shizuoka, TM3, Aomori, Kyo1, and 2489B and an FIV ancestor were Flag tagged at the C termini (the sequences are available upon request) and synthesized by the GeneArt gene synthesis service (Thermo Fisher Scientific). The obtained DNA fragments were inserted into the BamHI/SalI sites of the pDON-AI plasmid (TaKaRa). To construct the expression plasmids for a series of FIV Vif derivatives, the following primers were used: PT insert Fwd (TGG CCC TTC GTG AAC ATG TGG ATC AAG ACC GGC), PT insert Rev (ATG CGT TAA CGT CGA CTC ATT TGT CGT CGT CGT C), PT vector Fwd (GTC GAC GTT AAC GCA TGC), PT vector Rev (GTT CAC GAA GGG CCA TTC), TP insert Fwd (TGG CCC TTC GTG AAC ATG TGG ATC AAG ACC GGC), TP insert Rev (ATG CGT TAA CGT CGA CTC ATT TGT CGT CGT CGT C), TP vector Fwd (GTC GAC GTT AAC GCA TGC), TP vector Rev (GTT CAC GAA GGG CCA CTC), P-mu#1 Fwd (GAA CAT CTG CAA GGG CGG CGA GGT GTC CCA CGG ATG GGG ACC), P-mu#1 Rev (GGT CCC CAT CCG TGG GAC ACC TCG CCG CCC TTG CAG ATG TTC), P-mu#2 Fwd (GGC GAG CGG AAG ATC AAG GCC ACC CCC), P-mu#2 Rev (GGG GGT GGC CTT GAT CTT CCG CTC GCC), P-mu#3 Fwd (GCG GAA GAT CGA GAT CAC CCC CGT GAT G), P-mu#3 Rev (CAT CAC GGG GGT GAT CTC GAT CTT CCG C), P-mu#4 Fwd (GGC GAG ATC GAC CCC CAG AAG TGG TGC GGC), P-mu#4 Rev (GCC GCA CCA CTT CTG GGG GTC GAT CTC GCC), P-mu#5 Fwd (CCA TGC TGG CTT GTG GCA AAG AGG CCA AAG AAT GGC GGG GCT GC), P-mu#5 Rev (GCA GCC CCG CCA TTC TTT GGC CTC TTT GCC ACA AGC CAG CAT GG), P-mu#6 Fwd (GAT TCG TGT CCC CCT TCC GGA CCC CTG CC), P-mu#6 Rev (GGC AGG GGT CCG GAA GGG GGA CAC GAA TC), P-mu#7 Fwd (CTG GAA GTG ATC CAG AAC AAG CCC AAG CGG AAT CTG CTG TG), P-mu#7 Rev (CAC AGC AGA TTC CGC TTG GGC TTG TTC TGG ATC ACT TCC AG), P-mu#8 Fwd (GGA AGT GAT CCA GAA CAA GCC CAG CTG G), P-mu#8 Rev (CCA GCT GGG CTT GTT CTG GAT CAC TTC C), P-mu#9 Fwd (CCA GAG CAA GCC CAA GTG GAA TCT GCT G), P-mu#9 Rev (CAG CAG ATT CCA CTT GGG CTT GCT CTG G), P-mu#10 Fwd (GCA AGC CCA GCC GGA ATC TGC TGT GGA G), P-mu#10 Rev (CTC CAC AGC AGA TTC CGG CTG GGC TTG C), T-mu#1 Fwd (GAA CAT CTG CAT CGG CGG CGA GAT CAG CCC AGG CTG GGG ACC), T-mu#1 Rev (GGT CCC CAG CCT GGG CTG ATC TCG CCG CCG ATG CAG ATG TTC), T-mu#2 Fwd (GGC GAG CGG AAG ATC GAG GCC ACC CCC GTG ATG ATC), T-mu#2 Rev (GAT CAT CAC GGG GGT GGC CTC GAT CTT CCG CTC GCC), T-mu#3 Fwd (GGC GAG ATC GAC CCC AAG AAG TGG TGC GGC), T-mu#3 Rev (GCC GCA CCA CTT CTT GGG GTC GAT CTC GCC), T-mu#4 Fwd (CCA TGC TGG CCT GCG GCG TGC CCG CCA AAA AGT GGC GGG GCT GC), T-mu#4 Rev (GCA GCC CCG CCA CTT TTT GGC GGG CAC GCC GCA GGC CAG CAT GG), T-mu#5 Fwd (GAT TCG TGT CCC CCT ACC GGA CCC CCT GC), T-mu#5 Rev (GCA GGG GGT CCG GTA GGG GGA CAC GAA TC), T-mu#6 Fwd (CTG GAA GTG GTG CAG AGC AAG CCC AGC TGG AAC CTG CTG TG), T-mu#6 Rev (CAC AGC AGG TTC CAG CTG GGC TTG CTC TGC ACC ACT TCC AG), Petaluma vif His insert Fwd (ACC GAG CTC GGA TCC GCC ACC ATG AGC GAA GAG G), Petaluma vif His insert Rev (TGA GAT GAG TTT TTG TTC CAG CTC GCC GCT CCA CAG), TM219 vif His insert Fwd (ACC GAG CTC GGA TCC GCC ACC ATG AGC GAC GAG), TM219 vif His insert Rev (TGA GAT GAG TTT TTG TTC CAG CTC GCC GGT CCA CAG), C36 vif His insert Fwd (ACC GAG CTC GGA TCC GCC ACC ATG AGC GAA GAG G), C36 vif His insert Rev (TGA GAT GAG TTT TTG TTC CAG CTC GCC CAG CCA CAG), Shizuoka vif His insert Fwd (ACC GAG CTC GGA TCC GCC ACC ATG AGC GAA GAG G), Shizuoka vif His insert Rev (TGA GAT GAG TTT TTG TTC CAG CTC GCC GGT CCA CAG), vif His vector Fwd (GAA CAA AAA CTC ATC TCA GAA GAG GAT CTG AAT ATG CAT ACC GGT CAT CAT CAC CAT CAC CAT TGA GTC GAC GTT AAC GCA TGC), and vif His vector Rev (GGA TCC GAG CTC GGT ACC). The expression plasmids for a series of FIV Vif proteins, feline A3 derivatives, and pFP93 derivatives (pFIV*gagpol*Δ*vif*; a replication-incompetent *vif*-deficient FIV packaging construct derived from clone FIV-34TF10 [GenBank accession no. M25381], kindly provided by Eric M. Poeschla) (32) were constructed using the Gibson Assembly (New England BioLabs) or the GeneArt (Thermo Fisher Scientific) site-directed mutagenesis system. To construct a series of pFP93 derivatives, the following primers were used: PR-mutant insert Rev (GTA TTA CTT TTA CCT CTC CCT GGC AAT AAA TGT ATT TCT TTT G), PR-mutant vector Fwd (GAG AGG TAA AAG TAA TAC CAA C), PR-mutant vector Rev (CTA ATA AAA ATT TTA TAG GAT ATC CAT TTA CAA ATA TG), PR-TM2 insert Fwd (GAA ACT ATT GGA TTT ATA AAT TAT AAT ACA ATA GGT ACT ACC ACA AC), PR-TM2 insert Rev (CTT ATC AGA AAT TTG AGC CAT TAC CAA TCT TAT ATT AAA CTT AAT CAT G), PR-TM2 vector Fwd (GCT CAA ATT TCT GAT AAG ATT CC), and PR-TM2 vector Rev (ATT TAT AAA TCC AAT AGT TTC TCC TC).

### Cell culture and transfection.

HEK293T cells (CRL-11268; ATCC) were cultured in Dulbecco modified Eagle medium (Sigma-Aldrich) supplemented with 10% heat-inactivated fetal calf serum and antibiotics (Thermo Fisher Scientific). Transfection was performed using the Lipofectamine 2000 (Thermo Fisher Scientific) or the PEI Max (GE Healthcare) reagent according to the manufacturers' procedures. The anti-FIV activities of feline A3 proteins were evaluated as previously described (22). To analyze the dose-dependent effects of FIV PR on the parental A2Z2Z3 and the HSLH/IIII derivative (Fig. 7D to F), the expression plasmids (200, 400, 800, 1,200, and 1,800 ng) were cotransfected with pFP93 (800 ng) into HEK293T cells.

### Western blotting.

Western blotting was performed as previously described (22, 60, 68) using the following antibodies: anti-HA (clone 3F10; Roche), anti-Flag (rabbit polyclone OctA; Santa Cruz Biotechnology), anti-His (clone OGHis; Medical and Biological Laboratories), anti-FIV p27 (clone PAK3-2C1; Santa Cruz Biotechnology), anti-vesicular stomatitis virus G glycoprotein (anti-VSVg; clone P5DA; Roche), and anti-α-tubulin (TUBA; clone DM1A; Sigma-Aldrich). Five hundred microliters of viral supernatant was ultracentrifuged at 100,000 × *g* for 1 h at 4°C using a TL-100 instrument (Beckman), and the pellet was lysed with 1× SDS buffer. Transfected cells were lysed with radioimmunoprecipitation assay (RIPA) buffer containing a protease inhibitor cocktail (Roche). To quantify the amount of feline A3Z2Z3 and cleaved A3Z3 in the released viral particle (Fig. 7F), the band intensities of feline A3Z2Z3 and cleaved A3Z3 in the supernatant blot (a representative blot is shown in Fig. 7D) were quantified using Image Lab (v5.2) software (Bio-Rad).

### co-IP.

The expression plasmids for His-tagged FIV Vif (4 μg) and feline A3Z2Z3-HA (2 μg) were cotransfected into HEK293T cells in a 6-cm dish (Nunc) using the PEI Max reagent (GE Healthcare). The transfected cells were treated with the proteasome inhibitor MG132 (10 μM) at 24 h after transfection. The transfected cells were harvested 16 h later and resuspended in lysis buffer (150 mM NaCl and 1% Triton X-100 in phosphate-buffered saline [PBS]) containing a protease inhibitor cocktail (Roche) and MG132 (12.5 μM) at 4°C for 30 min. To perform the coimmunoprecipitation (co-IP) assay, cell lysates were mixed with magnetic beads conjugated to an anti-HA monoclonal antibody (clone M132-9; Medical and Biological Laboratories) and incubated at 4°C for 3 h. Then, the magnetic beads were washed with RIPA buffer and PBS and analyzed by Western blotting as described above.

### Statistical analyses.

The data are expressed as averages with the standard deviations (SDs), and statistically significant differences were determined using Student's *t* test and Bonferroni's multiple-comparison test.

### Accession number(s).

FIV *vif* sequences are available under GenBank/EMBL/DDBJ accession numbers LC177497 to LC177511 and LC179607 to LC179612.
